# Epigenetics of Sleep Disorders: An Emerging Field in Diagnosis and Therapeutics

**DOI:** 10.3390/diagnostics11050851

**Published:** 2021-05-10

**Authors:** Rene Cortese

**Affiliations:** Department of Child Health, Child Health Research Institute, School of Medicine, University of Missouri, Columbia, MO 65211, USA; rene.cortese@health.missouri.edu

The role of epigenetic regulation in sleep disorders is starting to be recognized. Major advances in our understanding of the role played by epigenetics in phenotypic expression of form and function have enabled development of new approaches and will likely open a new array of opportunities that may arise from the identification of epigenetic alterations that may provide high sensitivity and specificity for detecting, monitoring and predicting the outcomes of sleep disorders.

## 1. The Role of Epigenetics in Complex Pathologies

Epigenetics refers to the study of heritable changes in genome function that occur without a change in DNA sequence [1]. Epigenetic studies focus on three related molecular mechanisms: DNA methylation, histone code changes and non-coding RNAs (ncRNA) [2]. From a functional point of view, research on this field approaches issues such as how patterns of gene expression are transferred from one cell to its descendants, how gene expression changes during cell differentiation, and how environmental factors can alter the way genes are expressed, both temporarily and more permanently.

Assessment and evaluation of epigenomic profiles currently play a fundamental role in understanding, diagnosing and treating complex pathologies. Changes in epigenetic profiles at the cell or organismal level are among the most common molecular alterations in virtually all complex diseases, and may underlie both causal associations as well as constitute epiphenomena. Alterations of DNA methylation profiles have been recognized as an important, and in many cases essential, line of research to understand the pathophysiology of the specific condition of interest, as well as to develop diagnostic methods and therapeutic applications in cancer [3,4,5], diabetes [6], metabolic syndrome [7], atherosclerosis [8], inflammatory bowel disease [9], autoimmune diseases [10], psychiatric disorders [11], and neurodegenerative disorders [12]. As such, extrapolation of this extensive line of evidence to sleep disorders should not be viewed as particularly surprising.

An increasing number of environmental and physiological factors are now known to epigenetically regulate genes, including age [13], inflammation [14,15], stress [16], infection [17], cellular metabolism [18] and nutrition [19]. Remarkably, gestational epigenetic changes can also have long-term consequences [20]. Transient nutritional and other environmental stimuli during critical periods of prenatal and early postnatal development can affect the establishment or developmental maturation of gene-specific epigenetic profiles, thereby inducing permanent, partially reversible or reversible changes in gene expression [21].

Epigenetic alterations therefore represent very valuable biomarkers in molecular diagnosis, which are being consistently translated to clinical practice with great success. Furthermore, the potential of reversing epigenetic mechanisms provides opportunities for the development of targeted therapies and clinical interventions [22].

## 2. Sleep Disorders and Associated Morbidities. Need for Precision Medicine

Sleep disorders are extremely common conditions disturbing sleep patterns in adults and children [23,24]. They represent a wide spectrum of diseases, including sleep disruption due to environmental circumstance (e.g., living next to an airport or freeway), insomnia, restless leg syndrome, hypersomnia (e.g., Narcolepsy), circadian rhythm disorders, parasomnias and sleep disordered breathing (SDB; e.g., Sleep Apnea) to name a few [25]. Sleep disorders entail problems with the quality, timing and amount of sleep, which result in daytime distress and impairment in functioning [26].

Importantly, numerous and serious morbidities are associated with sleep disorders, virtually affecting every organ and system. Sleep disorders have been associated with the occurrence of metabolic [27], cardiovascular [28], and mood and cognitive diseases [29], as well as tumor aggressiveness and poor prognosis in cancer [30]. Moreover, gestational sleep disturbances can lead to adverse outcomes for both the mother and the child [31].

Currently, one of the major challenges in Sleep Medicine is to develop precise diagnostic approaches and tailored interventions leading to Precision Sleep Medicine. The need for biomarkers for diagnosis, monitoring, prognosis and the prediction of treatment effectiveness has been extensively recognized for the majority of sleep disorders and associated morbidities, including Obstructive Sleep Apnea [32,33], cognitive impairment [34], sleep loss [35], hypersomnia [36], behavior disorders [37], and circadian rhythm sleep-wake disorders [38]. Furthermore, the application of pharmacogenetic methods [39] and precision tools for personalized treatment selection [40] holds the promise for developing uniquely tailored approaches for managing sleep-disorder patients. Hence, currents research efforts are dedicated to the generation of knowledge and technological development towards these goals.

## 3. The Potential Contributions of Epigenetics in Precision Sleep Medicine

Since epigenetic alterations represent a common trait in the vast majority of complex diseases [41], they hold great potential as robust and precise biomarkers to be assessed in tissues and bodily fluids. A great deal of evidence and feasibility originates from cancer medicine, whereby epigenetics markers [42] and epigenetic-based therapies [43] have already been translated into clinical practice. Several features of epigenetically modified DNA species support their potential as early diagnostic biomarkers. First, the number of epigenetic aberrations in affected cells is significant and greatly exceeds the number of DNA sequence mutations [44,45]. Second, epigenetic DNA modifications occur in very early phases of disease development [46,47]. Third, many of the epigenetic DNA modifications are stable and can be detected in small amounts of DNA [22], which are evident advantages compared to RNA and proteomic strategies.

Besides the tremendous advances in cancer epigenetics, current research endeavors are ongoing towards the understanding of the epigenetic phenomena in the physiopathology of other complex diseases, and their potential applications in diagnostics and therapeutics. In particular, it is now accepted that epigenetic phenomena are key contributors in the homeostatic as well as circadian control of sleep [48]. A growing corpus of evidence demonstrates the fundamental role of epigenetic dysfunction in sleep apnea [49], insomnia [50] and sleep deprivation [51] and their comorbidities.

Based in this supportive, yet emerging evidence, studies on the application and clinical utility of epigenetic biomarkers and epigenetic-based therapies in sleep disorders are encouraged and should pave the way for major advances in the field. This Special Issue is dedicated to a critical appraisal of the current knowledge and to enable the presentation of novel findings derived from current research of the role of epigenetics in the pathophysiology of sleep disorders, the application of epigenetic markers for the diagnosis of sleep disorders and their comorbidities, and to the targeted potential of epigenetic interventions aimed at mitigating or resolving the adverse consequences of sleep disorders.

## Data Availability

Not applicable.
